# EnhancerAtlas 2.0: an updated resource with enhancer annotation in 586 tissue/cell types across nine species

**DOI:** 10.1093/nar/gkz980

**Published:** 2019-11-19

**Authors:** Tianshun Gao, Jiang Qian

**Affiliations:** 1 The Wilmer Eye Institute, Johns Hopkins School of Medicine, Baltimore, MD 21231, USA; 2 The Sidney Kimmel Comprehensive Cancer Center, Johns Hopkins School of Medicine, Baltimore, MD 21205, USA

## Abstract

Enhancers are distal *cis*-regulatory elements that activate the transcription of their target genes. They regulate a wide range of important biological functions and processes, including embryogenesis, development, and homeostasis. As more and more large-scale technologies were developed for enhancer identification, a comprehensive database is highly desirable for enhancer annotation based on various genome-wide profiling datasets across different species. Here, we present an updated database EnhancerAtlas 2.0 (http://www.enhanceratlas.org/indexv2.php), covering 586 tissue/cell types that include a large number of normal tissues, cancer cell lines, and cells at different development stages across nine species. Overall, the database contains 13 494 603 enhancers, which were obtained from 16 055 datasets using 12 high-throughput experiment methods (e.g. H3K4me1/H3K27ac, DNase-seq/ATAC-seq, P300, POLR2A, CAGE, ChIA-PET, GRO-seq, STARR-seq and MPRA). The updated version is a huge expansion of the first version, which only contains the enhancers in human cells. In addition, we predicted enhancer–target gene relationships in human, mouse and fly. Finally, the users can search enhancers and enhancer–target gene relationships through five user-friendly, interactive modules. We believe the new annotation of enhancers in EnhancerAtlas 2.0 will facilitate users to perform useful functional analysis of enhancers in various genomes.

## INTRODUCTION

As distal regulatory DNA elements, enhancers regulate the gene expression in a cell type-specific manner and function in a wide range of biological processes, including embryogenesis, development, homeostasis and diseases (1–3). With the development of Next-Generation Sequencing (NGS), multiple high-throughput experimental methods were designed to detect thousands of enhancers in different cell types (2–9). These methods can be classified into five categories: (i) chromatin immunoprecipitation and sequencing (ChIP-seq) of various transcription factors (TFs), specific mediator or cofactors, and specific histone modifications (10). The method was adopted to identify enhancer-related binding sites. The TFs often regulate gene expression by binding to the DNA regulatory elements (11). A specific TF, EP300, was a well-known enhancer marker. It was shown that over 75% of P300 binding sites were associated with enhancers and located far away from transcription start sites (TSSs) (12). Recent studies also revealed that the RNA polymerase II with the largest subunit POLR2A could move away from gene coding regions and bind to thousands of enhancers (4,12,13). (ii) The open chromatin regions identified by chromatin accessibility assays. Specifically, DNase I digestion coupled to sequencing (DNase-seq), transposase-accessible chromatin followed by sequencing (ATAC-seq), formaldehyde-assisted isolation and sequencing (FAIRE-seq) and micrococcal nuclease sequencing (MNase-seq) have been used to define transcriptional enhancers (8,9,14–16). (iii) Bi-directionally transcribed nascent enhancer RNAs (eRNAs). A large number of eRNAs detected by global run-on sequencing (GRO-seq) and cap-analysis gene expression (CAGE) often indicated a direct enhancer activity. (iv) High-throughput reporter assays, which were employed to quantitatively and directly detect the enhancer activities of thousands of DNA regulatory elements. Two representatives, STARR-seq (self-transcribing active regulatory region sequencing) and MPRA (massively parallel reporter assay), produced a library of reporter DNA sequence constructs as well as the unique tags or barcodes to assess the enhancer activities of tested regulatory regions (5,17). (v) Methods based on chromatin interactions, including Hi-C (18,19) and ChIA-PET (20). These approaches could identify enhancers from enhancer–enhancer or enhancer–promoter interactions. It was reported that ∼53% of chromatin interactions measured by the RNA polymerase II based ChIA-PET are enhancer-related (20).

While these methods are powerful to identify enhancers on a genome-wide scale, none of them are perfect in terms of sensitivity and specificity. For example, eRNAs only identify ∼25% of all 12 000 neuronal enhancers in the mouse genome (4). Furthermore, some methods, such as STARR-seq and GRO-seq, have only been successfully applied to certain species (e.g. *Drosophila* and *C. elegans*) (5,8,21). While many enhancer databases exist, such as SEdb, HACER, RAEdb, HEDD, DiseaseEnhancer, TiED, GeneHancer, SEA, DENdb and dbSUPER (22–31), none of them combined the datasets obtained from all different high-throughput approaches for enhancer annotation (Supplementary Figure S1). GeneHancer (25) integrated the enhancers from four different enhancer resources, including Ensembl, FANTOM, VISTA and ENCODE (2,32–34). The comparison of the enhancer databases showed that most databases utilized one or a few approaches for enhancer analysis (Supplementary Figure S1).

A comprehensive database is highly desirable for integrating enhancers from these genome-wide approaches for a better quality annotation. We developed a database, EnhancerAtlas (35), in which we combined the enhancer annotation from multiple pieces of experimental evidence and provided a set of analytic tools. Here, we present an updated version, EnhancerAtls 2.0, which includes a huge improvement from the previous version. It has three main improvements: (i) The new version expanded the enhancer annotation to nine species. In total, EnhancerAtlas 2.0 contained 13 494 603 enhancers based on 16 055 genome-wide profiling datasets (e.g. H3K4me1/H3K27ac, Dnase-seq/ATAC-seq, P300, POLR2A CAGE-seq, ChIA-PET, GRO-seq, STARR-seq and MPRA) in nine species. (ii) We improved the methods for combining multiple experimental datasets. (iii) New browse and new analytic tools were introduced to improve the web server.

## MATERIALS AND METHODS

### Data sources

The consensus enhancers in EnhancerAtlas 2.0 were identified based on twelve high-throughput experimental approaches, including P300 (12), Histone (10), POLR2A (13,21), TF-binding (11), DHS (or ATAC) (8,9), FAIRE (16), MNase-seq (14,15), GRO-seq (6), STARR-seq (5), CAGE (2), ChIA-PET (20) and MPRA (17). We manually downloaded 16 055 datasets, including processed or the raw sequencing data, from NCBI GEO datasets (36), ENCODE project portal at UCSC (32), Epigenome Roadmap (7) and FANTOM5 (2). The datasets in *Homo sapiens, Sus scrofa, Rattus norvegicus, Mus musculus, Gallus gallus, Danio rerio, Drosophila melanogaster, Caenorhabditis elegans* and *Saccharomyces cerevisiae* were mapped to hg19, susScr3, rn5, mm9, galGal4, danRer10, dm3, ce10 and sacCer3, respectively.

### Data processing for individual dataset/track

To build EnhancerAtlas 2.0, we collected 16 055 datasets. We first converted processed data into standard bed file based on their formats. The datasets with original bed, gff or narrowpeak format will be directly converted into standard bed using bedtools (37). We called peaks from the datasets with bedgraph format using macs2 module ‘bdgpeakcal’ (38). Finally, the dataset with bigwig format was firstly converted into bedgraph and then used for peak calling. The datasets in other genome build will be transformed into the right version by liftOver (39). We also removed the irregular datasets with a size <5 kb or >10 mb, which may contain too few or too many peaks. Also, the peaks overlapping with promoter, exon or CTCF-defined insulator regions were removed. The tools liftOver and bigWigToBedGraph (39) were downloaded from http://hgdownload.cse.ucsc.edu/admin/exe/linux.x86_64/.

We also called the peaks from raw sequencing data (Supplementary Figure S2). We summarized the parameters used for different types of datasets (Supplementary Table S1). We chose the parameters based on the dataset characterization and our experience. GRO-seq could detect 5-prime-capped RNAs that accurately mark active transcriptional regulatory elements (TREs) including enhancers (6,40). We used a GRO-seq specific calling tool, dREG, to get the candidate active enhancers with a bi-directional transcription (6). Note that the input plus and minus bigwig files for dREG were processed with no normalization by ‘RunOnBamToBigWig’ (41). The CAGE method had been successfully used to identify tens of thousands of eRNAs (2). We obtained the CAGE candidate enhancers in both human and mouse from the FANTOM5 project (http://fantom.gsc.riken.jp/5/datafiles/latest/extra/Enhancers/) (2).

RNA polymerase II based ChIA-PET detected the interactions between promoters and other regulatory regions including enhancers (20). We obtained the enhancer regions from the ChIA–PET interactions after filtering out the promoter and gene regions (Supplementary Tables S2 and S3). The MPRA data was collected from the RAEdb resource (29). In addition, we obtained STARR-seq datasets from the GEO Datasets (36).

### Generation of consensus track

We developed an unsupervised learning approach to weigh each track and combine them to determine the consensus enhancers (35). In this version, we improved the method by making a few adjustments of the method. To get the consensus track, we first normalized each individual track. The individual track usually includes several datasets. Especially for the ‘TF-binding’ track, it could contain dozens of datasets for different TFs. We normalized each dataset to make them comparable for combination. The normalization on each dataset or track was defined as:

$$\begin{equation*} {{s}}_{{i}}^{\rm{{\prime}}} = {{{s}}_{{i}}}\Bigg/\left( {\mathop \sum \nolimits_1^{{n}} \Big({{{s}}_{\rm{i}}}{{{l}}_{\rm{i}}}}\Big) \Bigg/\mathop \sum \nolimits_1^{{n}} {{{l}}_{{i}}}\right) \end{equation*}$$

where ${{{s}}_{{i}}}$ and ${{{l}}_{{i}}}{\rm{\ }}$are the fold enrichment and length of peak ${{i}}$ ($1 \le {{i}} \le {{n}}$), respectively. We filtered out peaks with length over 2500 bp in each dataset. If one track contains multiple datasets (e.g. multiple TFs), we merged the datasets with the area centering the average summit in the size of the average peak width (ASW) (42).

In our previous version, we used the Pearson Correlation Coefficient (PCC) to evaluate the correlations between two tracks across the whole genome region (35). To weight more on the enhancer regions, rather the largely non-enhancer regions in the genome, in the new version, we used the Jaccard index with intersection over union to assess the similarity based on the overlapping degree between two different tracks.

$$\begin{equation*}\ {J_{{A_i}{A_j}}} = \frac{{Nu{m_{{A_i}\mathop \cap {A_j}}}}}{{Nu{m_{{A_i}\mathop \cup {A_j}}}}}\ \end{equation*}$$

where $Nu{m_{{A_i}\mathop \cap {A_j}}}$ represents the number of overlapped regions between tracks ${A_i}$ and ${A_j}$ while $Nu{m_{{A_i}\mathop \cup {A_j}}}$ means the number of union regions. Given a tissue/cell type with $m$ tracks, we calculated the similarities of all combinations of any two tracks and put them into a matrix as following:

$$\begin{equation*}\left[ {\begin{array}{@{}*{3}{c}@{}} {\begin{array}{@{}*{3}{c}@{}} {{{{J}}_{{{A_1}}{{A_1}}}}}& \cdots &{{{{J}}_{{{A_1}}{{A_t}}}}}\\ \vdots &{\rm{\ }}&{{\rm{\ }} \vdots } \end{array}}&{\begin{array}{@{}*{2}{c}@{}} \cdots &{{{{J}}_{{{A_1}}{{A_m}}}}}\\ {\rm{\ }}&{{\rm{\ }} \vdots {\rm{\ }}} \end{array}}\\ {\begin{array}{@{}*{3}{c}@{}} {{{\rm{J}}_{{{A_t}}{{A_1}}}}}& \cdots &{{{{J}}_{{{A_t}}{{A_t}}}}}\\ {{\rm{\ }} \vdots }&{\rm{\ }}& \vdots \\ {{{{J}}_{{{A_m}}{{A_1}}}}}& \cdots &{{{{J}}_{{{A_m}}{{A_t}}}}} \end{array}}&{\begin{array}{@{}*{2}{c}@{}} \cdots &{{{{J}}_{{{A_t}}{{A_m}}}}}\\ {\rm{\ }}& \vdots \\ \cdots &{{{{J}}_{{{A_m}}{{A_m}}}}} \end{array}} \end{array}} \right]\end{equation*}$$

For any track ${{A_t}}$, we calculated its weight:

$$\begin{equation*}\ {{{w}}_{{t}}} = \frac{{\mathop \sum \nolimits_{{{j}} = 1}^{{m}} {{{J}}_{{{A_t}}{{A_j}}}}}}{{\mathop \sum \nolimits_{{{j}} = 1,{{k}} = 1}^{{m}} {{{J}}_{{{A_k}}{{A_j}}}}}}{\rm{\ \ }}\left( {{{j}},{{k}} \in \left[ {1,{{m}}} \right],{{j}} \ne {{t}},{{j}} \ne {{k}}} \right)\end{equation*}$$

In addition, we set that each peak in the merged profile must be supported by at least 50% of tracks. The signal value for each combined peak was determined as:

$$\begin{equation*}\ Scor{e_{combined}} = \frac{{\mathop \sum \nolimits_{t = 1}^m {w_t}{L_t}Scor{e_{A_t}}}}{{{L_{combined}}}}\ \end{equation*}$$

where ${L_t}$ and ${{{L}}_{combined}}{\rm{\ }}$ means the length of relative peak in track ${{A_t}}$ and the length of combined peak in the merged consensus track, respectively.

### Enhancer–gene interactions

We developed an algorithm, an Enhancer And Gene based Learning Ensemble method (EAGLE), to identify Enhancer–Gene (EG) interactions (43). The method is based on six features, including correlation between enhancer activity and gene expression across cell types, gene expression level of target genes, genomic distance between an enhancer and its target gene, enhancer signal, average gene activity in the region between the enhancer and target gene and enhancer–enhancer correlation. These genomic features were derived from enhancers and gene expression datasets from the same cell type. Therefore, the method could be widely used in different tissue/cell types. We used ChIA-PET (20) and/or Hi-C (18,19) as the gold standards to define the training datasets and built three prediction models for human, mouse and fly, respectively (Supplementary Figure S3). Applying EAGLE to these three species, we identified 7 680 203, 7 437 255 and 317 588 EG interactions involving 31 375, 43 724 and 12 766 genes, 138 547, 177 062 and 40 321 enhancers across 89, 110, and 7 tissue/cell types in mouse, human, and fly respectively (43). We will provide the enhancer-gene relationships for the other species when the genomic interaction datasets (e.g. ChIA–PET and Hi-C) become available for these species.

### Implementation of database

The EnhancerAtlas 2.0 runs on a Linux platform based on Apache-Tomcat-MySQL-PHP-HTML5-JavaScript-Perl and can be used on Windows, Mac and Linux. Specially, we designed a genome browser to display the coordinates and signals of individual datasets as well as consensus track in specific cells. If the enhancer-gene interactions are available for a particular cell type, they will also be displayed. The visualization was implemented using the HTML5 <canvas> element and a drawing module in JavaScript. A two-handle slider widget in the genome browser was set to zoom in or out the genome area. We provided several useful analytic tools so that the users can compare enhancers across species or predict enhancers in their own datasets.

## RESULTS

### Statistics

EnhancerAtlas 2.0 included 13 494 603 annotated consensus enhancers based on 16 055 datasets in 586 tissue/cell types across nine species. The datasets have 12 major data types (tracks) (Table 1). The number of datasets, tracks and enhancers are also summarized in each species (Supplementary Tables S2–S10). For some species (e.g. *H. sapiens*, *M. musculus*, *D. melanogaster* and *C. elegans*), we determined the consensus enhancers with at least three tracks for each tissue/cell type (Supplementary Tables S2–S5). For the remaining species, we have at least two tracks in each cell type (Supplementary Tables S6–S10). If only two tracks are available for a particular cell type, we require that the consensus enhancer must be supported by both tracks. We also predicted 7 680 203, 7 437 255 and 317 588 enhancer–target gene interactions in human, mouse and fly, respectively. We plan to predict the interactions for the other species when the Hi-C (18,19) and/or ChIA-PET (20) datasets become available.

**Table 1.** Summary of the numbers for tissue/cells, consensus enhancers, total datasets and the datasets of 12 tracks in nine species.

	Tissue/cells	Enhancers	Datasets	P300	POLR2A	Histone	TF-binding	DHS	FAIRE	MNase	GRO	CAGE	MPRA	STARR	CHIA-PET
*Homo sapiens*	277	6 031 402	8005	132	696	1580	4159	1113	116	56	31	83	5	10	24
*Mus musculus*	241	6 198 364	5838	102	451	1533	2930	592	60	91	24	47	0	0	8
*Drosophila melanogaster*	21	294 158	801	0	101	85	396	96	30	53	22	0	0	17	0
*Caenorhabditis elegans*	9	53 060	954	0	69	36	677	150	0	16	6	0	0	0	0
*Danio rerio*	15	324 595	117	0	2	30	37	42	4	2	0	0	0	0	0
*Rattus norvegicus*	11	267 542	101	1	6	37	48	8	0	0	1	0	0	0	0
*Gallus gallus*	3	248 792	35	0	6	8	15	6	0	0	0	0	0	0	0
*Sus scrofa*	2	71 851	11	1	0	2	4	0	0	1	3	0	0	0	0
*Saccharomyces cerevisiae*	7	4839	197	0	24	17	115	7	6	27	0	0	0	0	0
Total	586	13 494 603	16 055	236	1355	3324	8382	2014	216	246	87	130	5	27	32

### Database search

**Figure 1.**
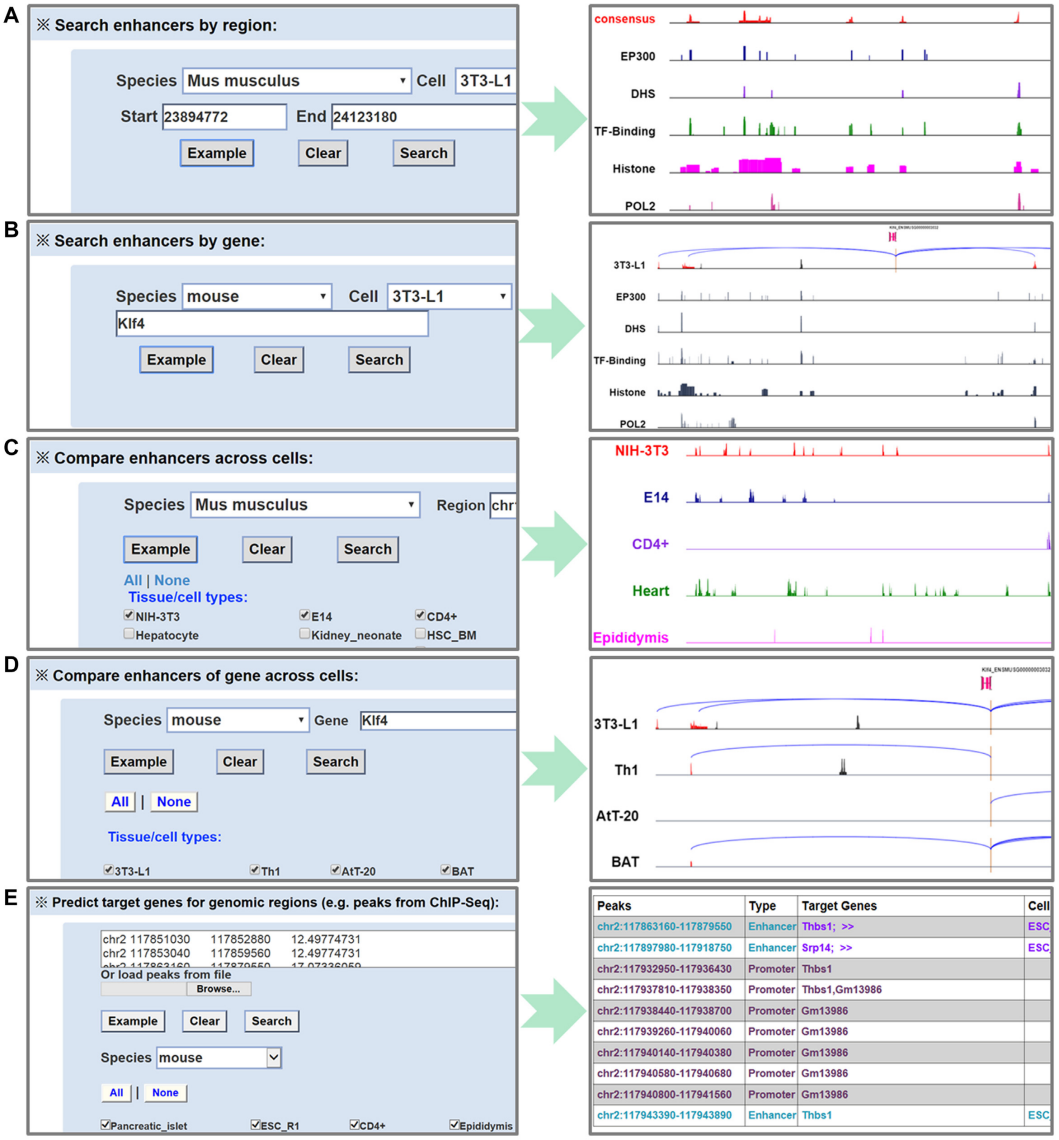
Search options. (**A**) Enhancers in a given genome region can be identified. (**B**) The enhancers that regulate an input gene could be searched. (**C**) Users can search and compare different enhancers in a set of tissue/cells. (**D**) The enhancers regulating the same gene in the different cell types can also be compared. (**E**) Users can identify the promoters, enhancers in different tissue/cells, and the potential target genes for these enhancers with a given set of peaks of interest.

EnhancerAtlas 2.0 was constructed in a user-friendly way. It provided easy-to-use web interfaces for users to search, browse and download different types of enhancers and enhancer-gene interactions in different species. We provided five web-based analytical tools to query and visualize the enhancers and enhancer–gene interactions: (i) search enhancers by region, (ii) search enhancers by gene, (iii) compare enhancers across cells, (iv) compare enhancers of gene across cells, (v) predict enhancers and target genes for custom datasets. Users can search the enhancers by region in any tissue/cell of any species (Figure 1A).

Users can search the enhancers that regulate a particular gene of interest using the second search option (Figure 1B). The input of gene name or ID can be in many formats, such as Ensembl, EMBL, UCSC, PDB, FlyBase, RefSeq and UniProt (33,44–49). A genome browser will be provided for users to visualize the enhancer–gene interactions in the genome. Users could also compare enhancers across different tissue/cell types to identify conserved or cell type-specific enhancers using the third search option (Figure 1C). A gene could be regulated by different enhancers in different tissue/cells. The fourth search tool will let users to visualize the different enhancers that regulate the input gene in different tissue/cells (Figure 1D). Users can click the cell names to access the detailed track information for each individual cell. Users can also click ‘show the details’ or ‘download enhancers associated with the gene’ to get the list of enhancer-gene interactions in all selected cells and obtain relevant enhancers (Supplementary Figure S4). We also designed a module to help users to identify the promoters, potential enhancers and the target genes of enhancers from a set of peaks (e.g. obtained from a ChIP-seq or ATAC-seq dataset) (Figure 1E).

### Enhancer browser

**Figure 2.**
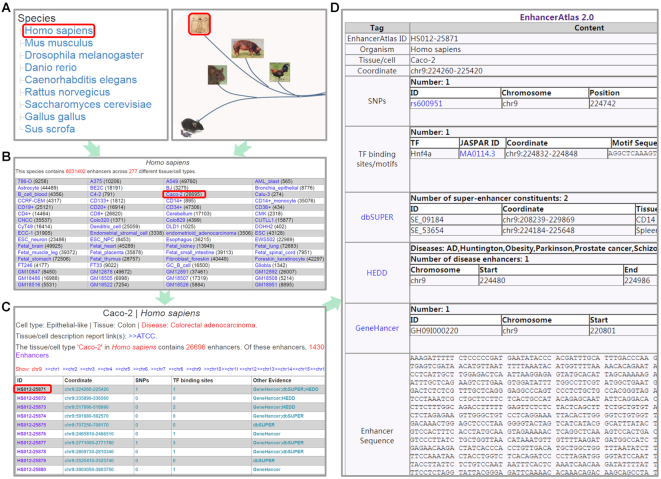
Enhancer browser. (**A**) Users can select a species to browser all the tissue/cells by clicking the name or image of this species. (**B**) A tissue/cell browse page for the selected species could facilitate users to browse any tissue/cell of its enhancers. (**C**) The enhancer summary was provided for the selected tissue/cell. (**D**) A summary table of the detailed information for each enhancer could be obtained from (**C**).

We also provide a browser page for each enhancer. Users can select the species, cell type, chromosome and a particular enhancer, and the database will generate a summary table, which includes coordinate of the enhancer, GWAS SNPs (50) within the enhancer, TF binding motifs from JASPAR (51), associated super-enhancer, related disease and enhancer sequence (Figure 2).

## CONCLUSIONS

EnhancerAtlas 2.0 has a great improvement from version 1.0. It annotated 13 494 603 consensus enhancers in 586 tissue/cell types from 12 high-throughput technologies across nine species. We believe this is the most comprehensive enhancer database that includes the largest number of enhancer-related datasets. The database has the following advantages. First, it provides enhancer consensus annotation for ∼600 tissue/cell types, which represent the reliable enhancer annotation. Second, it provides useful analytic tools that users can search, compare and download the enhancers of interest. Third, we also provided the potential enhancer–target gene interactions using a newly developed method, EAGLE (43). The method outperformed IM-PET (52), which we used to predict enhancer–target gene interactions in our previous version of database. Finally, we optimized the search functions in the website, which increased the convenience for users to search, query and browse our database. For the future development, we plan to provide more relevant information of the enhancers such as evolutionary conservation across species and associated diseases (24,27,28,30,31).

## DATA AVAILABILITY

All the data can be downloaded in http://www.enhanceratlas.org/downloadv2.php.

## Supplementary Material

gkz980_Supplemental_Files
