# Supplementary material for: EnhancerAtlas 2.0: an updated resource with enhancer annotation in 586 tissue/cell types across nine species
Source: Nucleic Acids Res. 2019 Nov 19;48(D1):D58–64. doi: 10.1093/nar/gkz980 (PMC7145677; doi:10.1093/nar/gkz980)
Supplement: gkz980_Supplemental_Files [file gkz980_supplemental_files.zip › EnhancerAtlas2_supple_revision.docx]

**Supplementary Data:**

**EnhancerAtlas 2.0: an updated resource with typical enhancer annotation in 586 tissue/cell types across nine species**

Tianshun Gao^1^, Jiang Qian^1,2^*

^1^ The Wilmer Eye Institute, Johns Hopkins School of Medicine, Baltimore, MD 21231, USA.

^2^ The Sidney Kimmel Comprehensive Cancer Center, Johns Hopkins School of Medicine, Baltimore, MD 21205, USA


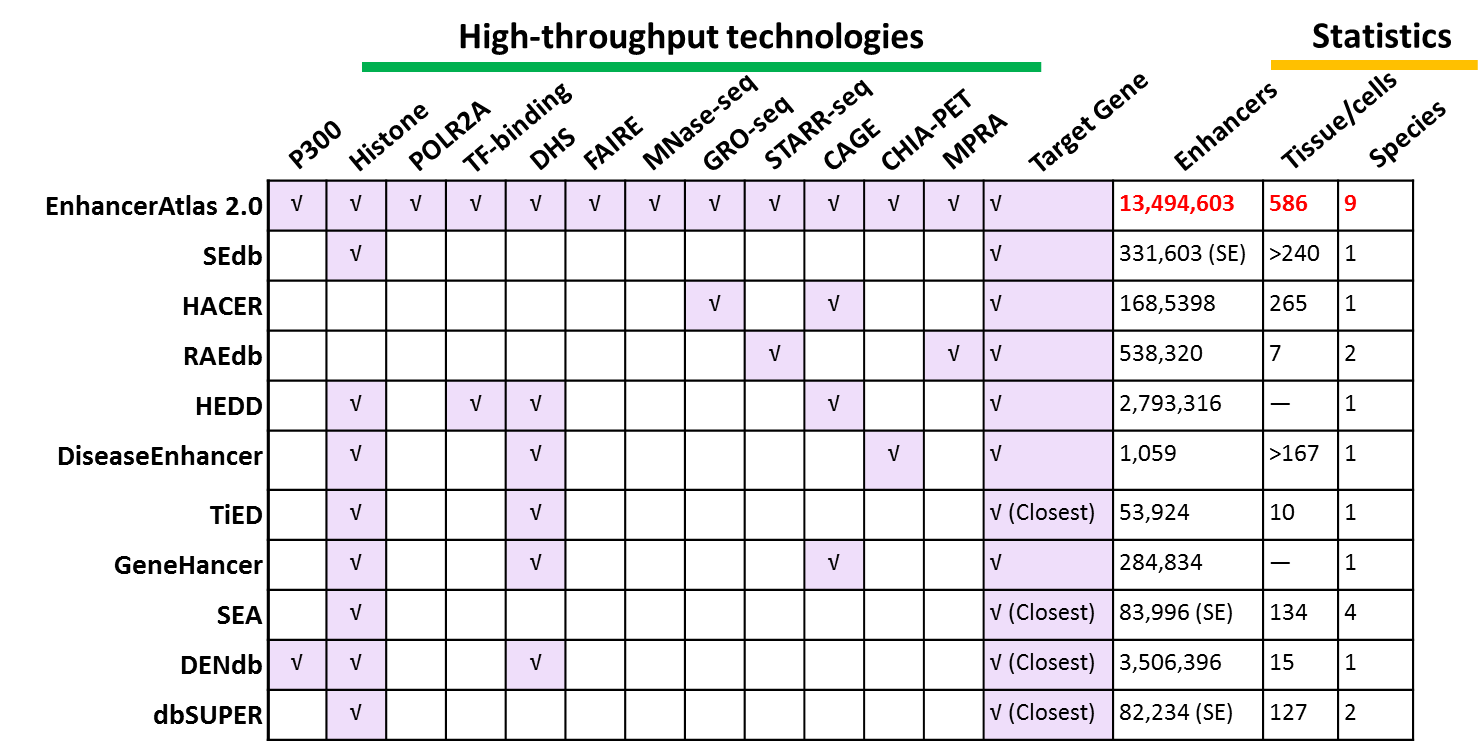


**Figure S1.** Comparison among EnhancerAtlas 2.0 and other databases. Note that the “enhancer” used in “Statistics” for SEdb, SEA, and dbSUPER meant the “super-enhancer (SE)”. Some databases simply used the “closest” gene from enhancer as the target gene, while EnhancerAtlas 2.0 used six discriminative genomic features to identify the target gene.

**
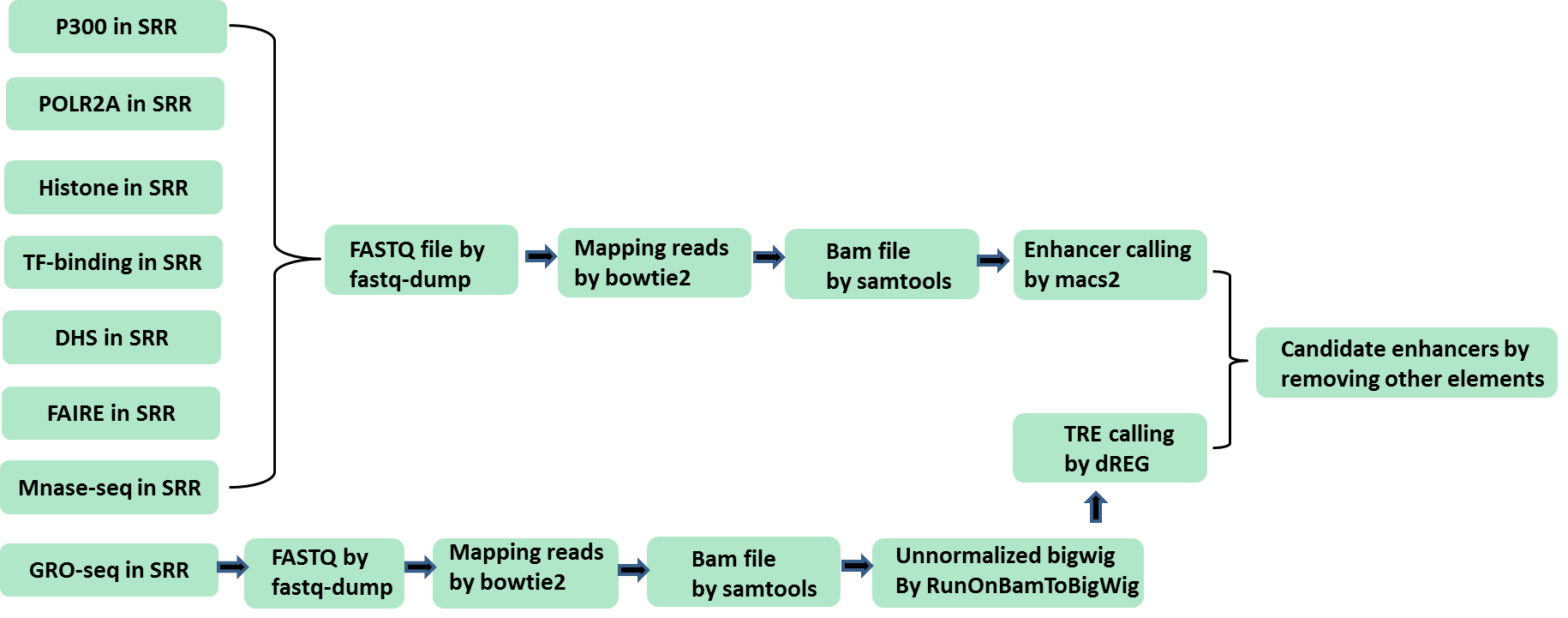
**

**Figure S2.** Overview of candidate enhancer peak calling from the raw sequencing datasets.


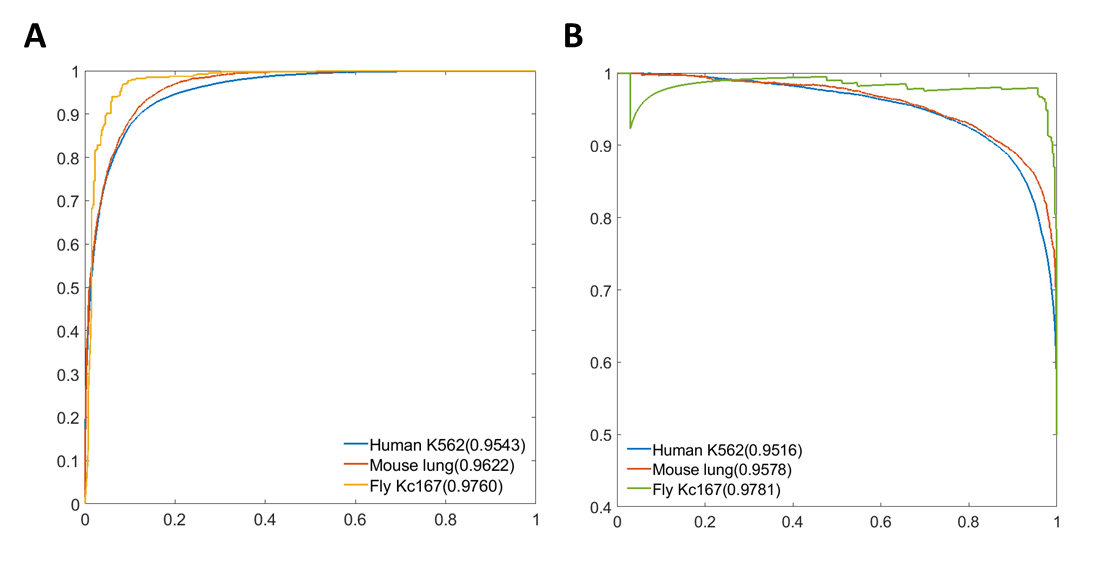


**Figure S3** Performance of trained models by 10-fold cross-validation in three species. (A) High AROCs (Area under the Receiver-Operating Curves) of 0.9543, 0.9622 and 0.9760 in human, mouse and fly, respectively. (B) High performances of three models by Precision-Recall (PR) curve plot.

**
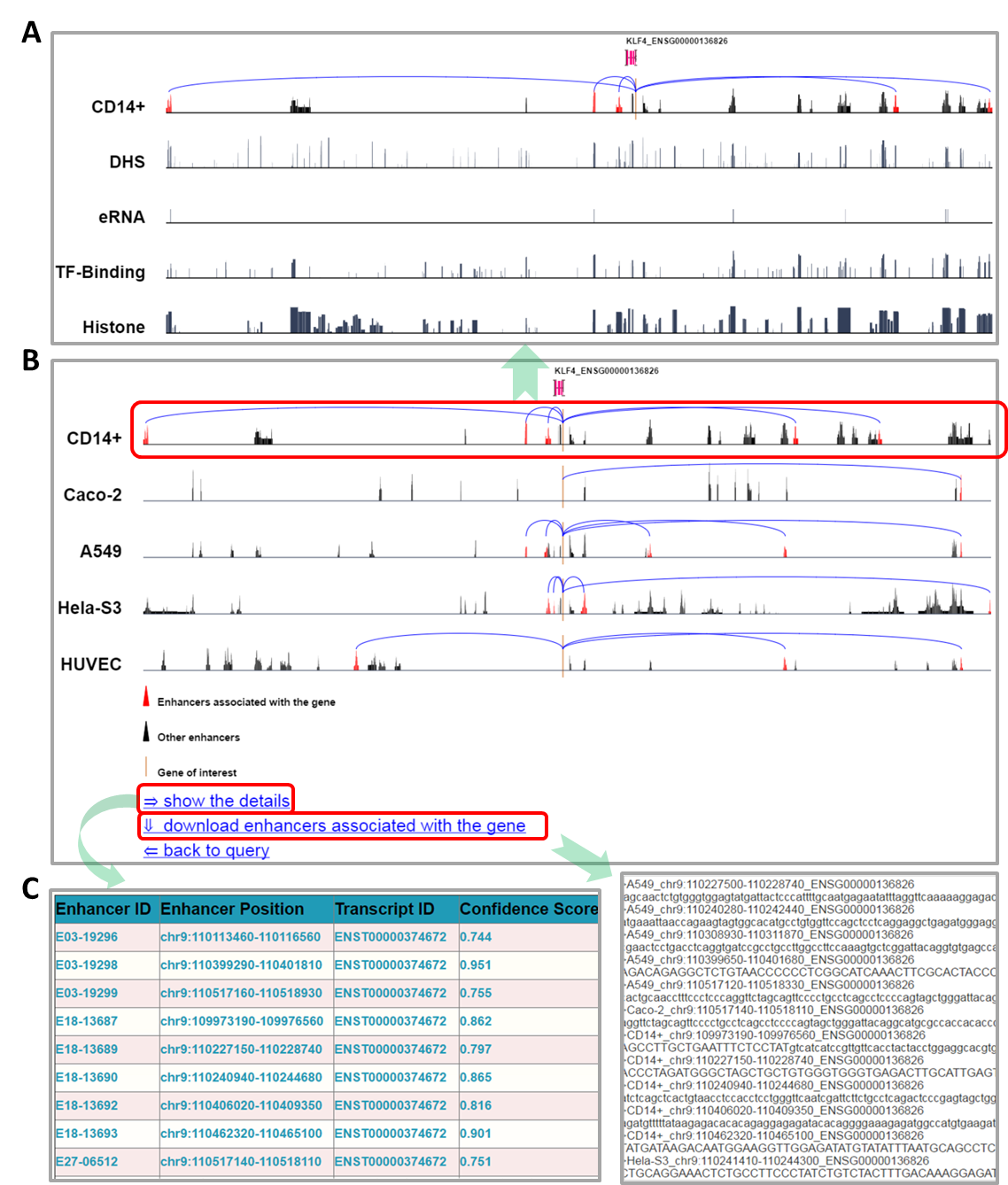
**

**Figure S4.** Genome browse page. (A) Enhancer-gene interaction display of individual tissue/cell after clicking relative tissue/cell in (B). (B) Genome browse page for enhancer-gene interactions across many tissue/cells, with the links into other resources. (C) Enhancer-gene interaction list and the gene related enhancer sequences when clicking the links.
